# Toward a recursive process model of problematic short-form video use: the Reinforcement–Narrowing–Shift framework

**DOI:** 10.3389/fpsyg.2026.1871950

**Published:** 2026-10-30

**Authors:** Wenci Ge, Yanli Hua, Yihuan Yuan, Shengxuan Li, Dongming Li

**Affiliations:** 1 School of Languages and Culture, Hebei GEO University, Shijiazhuang, China; 2 College of Life Sciences, Hebei Normal University, Shijiazhuang, China

**Keywords:** algorithmic recommendation, cognitive-affective shift, emotion regulation, informational narrowing, multilevel intervention, problematic short-form video use

## Abstract

Short-form video (SFV) platforms have become a central component of contemporary digital media use. Although research has examined problematic short-form video use (PSVU), existing accounts often emphasize addictive-like symptoms or individual self-control while giving less attention to the exposure environment that recommendation systems continually update. This structured integrative conceptual review develops a recursive Reinforcement–Narrowing–Shift (RNS) framework to explain how PSVU may emerge and become self-maintaining. The framework proposes that interactions between platform affordances—including auto play, infinite scroll, full-screen presentation, rapid switching, and fine-grained behavioral feedback—and user vulnerabilities can reinforce repeated engagement. Under identifiable boundary conditions, including engagement-dominant optimization, stable behavioral signals, weak diversity constraints, and predominantly passive consumption, repeated engagement may contribute to multidimensional informational narrowing. We distinguish exposure-level narrowing in content and affective diversity from experience-level narrowing in cognitive demand and temporal horizons. These changes may be translated into cognitive-affective shifts through processing fluency and metacognitive erosion, affective habituation and escalation, and attentional capture and automaticity, thereby increasing renewed engagement. The framework does not assume that personalization necessarily narrows exposure: exploration, serendipity objectives, active user search, changing preferences, and external life events can stabilize, diversify, or interrupt the cycle. We develop nine falsifiable propositions, organize them into feasible research modules, and identify executive control, developmental stage, and cultural-institutional context as moderators. RNS is presented as a new platform-sensitive research framework rather than an extension of an existing clinical model, offering testable directions for longitudinal, experimental, computational, and intervention research.

## 1 Introduction

Short-form video (SFV) platforms, such as TikTok, Douyin, and YouTube Shorts, have become a central part of contemporary digital media use across age groups and cultures (Montag et al., 2021). In this review, an SFV environment refers to the integrated affordance ecology created by brief audiovisual units, full-screen single-item presentation, rapid one-swipe transitions, auto play or continuous feeds, fine-grained behavioral sensing, and continuously updated recommendation. This environment is therefore more than a video format: it couples interface design, behavioral reinforcement, and algorithmic selection. Recommendation systems update content streams using signals such as dwell time, completion, re watches, skips, pauses, likes, comments, shares, follows, and prior histories (Quan et al., 2024), while auto play, infinite scroll, and low-friction switching make continuation easier than deliberate stopping (Zhao and Wagner, 2023).

These affordances create a media environment in which content is short, emotionally salient, personalized, and continuously available. SFV platforms therefore function not only as sources of entertainment but also as channels for information exposure, social interaction, identity expression, and mood regulation in daily life (Omar and Dequan, 2020; Scherr and Wang, 2021; Chen et al., 2024). The expanding role of SFV platforms has raised concerns that routine use may, for some users, develop into problematic short-form video use (PSVU). In this article, PSVU is conceptualized as dysregulated SFV engagement rather than as high-frequency use alone or as a formal clinical diagnosis. It refers to a pattern characterized by difficulty disengaging, cue-triggered checking, reliance on SFVs for affect regulation, reduced reflective control, and interference with everyday functioning. This definition is consistent with broader process models of behavioral addictions, which emphasize impaired control, cue-reactivity and craving, habitual responding, diminished inhibitory control, and functional impairment rather than high-frequency use alone (Brand et al., 2019).

Recent reviews and empirical studies suggest that PSVU is associated with demographic, psychological, personality-related, behavioral, social, motivational, and platform-induced factors (Zhang et al., 2019; Qin et al., 2022; Zhang D. et al., 2024; Zhan and Zhu, 2025). Contemporary behavioral-addiction research further emphasizes functional impairment, impaired control, and persistence despite adverse consequences rather than high frequency alone, while also cautioning against treating every intensive digital behavior as a formal disorder (Brand et al., 2025). Research relevant to PSVU spans problematic digital media use, algorithmic curation and selective exposure, and the cognitive-affective consequences of rapid and emotionally salient media use. These literatures have rarely been integrated into a model that treats the user's informational environment as both an outcome of earlier behavior and a cause of later behavior.

To address this gap, this structured integrative conceptual review develops a recursive Reinforcement–Narrowing–Shift (RNS) framework for understanding PSVU. The framework synthesizes evidence on SFV affordances, behavioral reinforcement, algorithmic recommendation, informational diversity, cognitive-affective processes, and problematic digital engagement. Its distinctive focus is the recursively updated informational environment produced by interactions between user behavior and recommendation systems.

This article employs a structured integrative conceptual-review approach. A supplementary literature search, completed on 1 October 2026, used iterative searches of PubMed, Cross ref, and Google Scholar. Search concepts combined terms for short-form video, TikTok, Douyin, reels, problematic use, addiction-like use, auto play, infinite scroll, intermittent or variable reinforcement, recommendation systems, personalization, diversity, serendipity, filter bubbles, cognitive load, processing fluency, metacognition, automaticity, data donation, and digital intervention. We also conducted backward and forward citation chaining from major process models and from studies identified by the reviewers. The supplementary search log reports the core concept blocks, and screening principles.

Sources were included when they directly examined SFV use or clarified a mechanism required by the RNS framework; adjacent research on social media, smartphones, gambling, gaming, recommendation systems, and cognitive psychology was retained only when its transfer was explicitly qualified. We considered supportive, mixed, null, and countervailing evidence, including work showing that personalization can introduce novelty or diversity rather than narrowing exposure. Because terminology, platforms, and methods are heterogeneous, the review does not claim systematic-review exhaustiveness or permit pooled effect estimates. Its purpose is to develop and delimit a falsifiable process model while making the evidential status of each pathway visible (Torraco, 2005; Gilson and Goldberg, 2015).

This review makes three main theoretical contributions to research on problematic digital media use and algorithm-driven engagement. First, it proposes a new platform-sensitive framework in which algorithmically mediated informational narrowing may connect reinforced engagement with later cognitive-affective change. Second, it clarifies the distinction between filter bubbles, echo chambers, information cocoons, and informational narrowing. Third, it links the proposed mechanisms to multilevel intervention targets. Together, these contributions conceptualize PSVU as a dynamic and potentially interruptible process while keeping RNS distinct from established clinical process models.

## 2 Platform architecture and the front-end mechanisms of PSVU

This section focuses on the early mechanisms through which ordinary SFV use may become difficult to control. The aim is to explain how platform design, reinforcement processes, habit formation, and user vulnerability interact. These mechanisms help explain why some users can disengage easily, whereas others find it increasingly difficult to stop. In this article, these processes are termed front-end mechanisms because they operate at the early stages of problematic engagement, before the full recursive cycle of informational narrowing and cognitive-affective shift becomes consolidated.

### 2.1 Low-friction continuation and reinforcement

SFV platforms minimize the action required to continue. Autoplay, infinite scroll, full-screen viewing, and one-swipe switching remove repeated choice points and make continuation the default (Zhao and Wagner, 2023; Xiong et al., 2024). These affordances should not be treated as addictive in isolation. Their relevance is conditional: they are expected to exert stronger effects when users are fatigued, distressed, bored, or weakly committed to another goal, and when platform defaults provide few salient stopping cues. Repeated responses in stable contexts can then become increasingly automatic (Wood and Rünger, 2016).

Intermittent reinforcement provides a complementary account. Engaging videos occur unpredictably among less engaging items, so each swipe may produce humor, useful information, social validation, surprise, or emotional relief. This variable sequence can sustain sampling even when average satisfaction is modest (Liao, 2024). The mechanism remains incompletely tested in SFV settings: unpredictability, novelty, content quality, and algorithmic adaptation are often confounded. RNS therefore treats intermittent reward as a testable contributor to continued engagement rather than a settled causal explanation.

These affordances may intensify the proposed bridging mechanisms relative to longer-form or choice-based media because the interval between exposure, behavioral feedback, and the next recommendation is compressed. Full-screen single-item presentation reduces competing visual cues, one-swipe transitions multiply cue–response cycles within a session, and completion or skipping provides rapid signals for feed updating. This comparative claim is conditional rather than deterministic: direct experiments isolating auto play or intermittent reward in SFV environments remain limited, and similar mechanisms operate in other digital media. Existing evidence on binge-scrolling, flow, time distortion, and view-time-oriented recommendation nevertheless supports testing whether higher feedback frequency accelerates automaticity, time-estimation error, and feed convergence (Quan et al., 2024; Zhao and Wagner, 2023; Park and Jung, 2024; Yang et al., 2024).

### 2.2 From intentional use to habitual engagement

SFV use often begins with explicit goals such as relaxation, entertainment, information seeking, social connection, or mood regulation (Scherr and Wang, 2021; Chen et al., 2024). With repetition, boredom, fatigue, loneliness, stress, procrastination, bedtime routines, or the visible app icon may become cues for opening the platform. Dual-process accounts distinguish reflective, goal-directed action from faster associative responses (Strack and Deutsch, 2004; Turel and Bechara, 2016). Incentive-sensitization theory further distinguishes cue-triggered “wanting” from experienced “liking” (Berridge and Robinson, 2016). Applied cautiously outside substance addiction, this distinction suggests a testable SFV pattern: users may continue checking or scrolling even when enjoyment has declined. The relevant outcome is therefore not duration alone but a growing mismatch among intention, evaluation, and realized behavior.

### 2.3 User-level vulnerability

Platform design does not affect all users in the same way. Some users can engage with SFVs without losing control, whereas others may be more vulnerable. PSVU is therefore better understood as an interaction between platform conditions and user characteristics (Brand et al., 2019; Xiong et al., 2024). Affect regulation is one important pathway. SFV platforms provide rapid access to novelty, distraction, humor, emotional stimulation, and social cues (Xiao et al., 2023). These features make the platform attractive during negative affect, boredom, or fear of missing out (Huang et al., 2022; Mu et al., 2022; Chen et al., 2024; Zhan and Zhu, 2025). The core issue does not lie in users' pursuit of emotional relief. Instead, problems emerge when SFV use becomes the primary means of regulating negative emotional states. In that case, users may increasingly rely on rapid, immediately available distraction and have fewer opportunities to practice slower and more adaptive emotion-regulation strategies (Cheng et al., 2023; Nguyen et al., 2025).

Executive control is another important factor. Working memory, inhibitory control, attentional regulation, and cognitive flexibility help users resist cues, stop ongoing behavior, and stay focused on longer-term goals (Diamond, 2013; Brand et al., 2019; Turel and Bechara, 2016). Users with weaker executive control may find it harder to stop watching. They may also be more likely to check the platform repeatedly or stay longer in personalized and emotionally engaging feeds (Xie et al., 2023; Xu et al., 2024; Liu et al., 2025).

Beyond these cognitive regulatory capacities, stable individual differences in personality traits also contribute to users' SFV engagement and problematic use patterns. Impulsivity can amplify the appeal of immediate rewards. Boredom proneness may heighten attraction to rapidly changing video content. Neuroticism may drive SFV engagement for emotional reassurance, distraction, or mood regulation (Cheng et al., 2023). Lower conscientiousness may be associated with weaker adherence to planned routines and greater interference of SFV use with study, work, or sleep (Jiang and Yoo, 2024; Xiong et al., 2024; Zhan and Zhu, 2025). These personality traits do not independently determine behavioral outcomes. Instead, they help explain why similar platform designs can produce divergent usage patterns across individuals (Hong et al., 2025; Zhang L. et al., 2024). Developmental stage also matters. Adolescents and younger users may be more sensitive to immediate rewards while self-regulatory control is still developing (Casey et al., 2008). For these users, a platform that combines fast feedback, social validation, emotional stimulation, and easy continuation may be especially powerful in shaping habits. Emerging, largely correlational neurocognitive evidence suggests that PSVU is associated with altered reward processing and reduced executive control, although causal direction remains uncertain (Gao et al., 2025; Zhang and Li, 2025). Recent cross-sectional studies link short-video addiction or intensive use to reduced attention control and poorer perceived writing competence (Ye et al., 2025; Al-Leimon et al., 2025; Chen et al., 2026). Other studies associate short-video addiction or problematic use with weaker self-regulated learning and learning engagement, lower learning motivation and wellbeing, and greater perceived learning ineffectiveness (Ye et al., 2022, 2023, 2024; Li et al., 2024; Mao and Liao, 2025). These findings support the relevance of cognitive-regulatory and functional outcomes to RNS, but their cross-sectional designs do not establish causal direction, directly measure recommendation exposure, or test the full recursive sequence.

Taken together, these front-end mechanisms explain how SFV engagement may become repetitive, cue-triggered, and difficult to regulate. However, they do not fully explain why problematic use becomes self-maintaining over time. The next section builds on these initiation mechanisms by examining how they become consolidated through user-algorithm interaction.

## 3 The recursive Reinforcement–Narrowing–Shift (RNS) framework

Building on the front-end mechanisms described in Section 2, this section develops a recursive framework explaining how PSVU may become self-maintaining after initial engagement has been established. The framework centers on the interaction of reinforcement-driven engagement, algorithmically mediated informational narrowing, cognitive-affective shifts, and renewed problematic engagement. Figure 1 presents these recursive components and the processes linking them, together with moderating and countervailing conditions. The feedback pathway indicates how later cognitive-affective shifts and renewed engagement may reactivate earlier reinforcement processes. The RNS framework was developed through the structured integrative review described above. Its components were synthesized from evidence on platform affordances, reinforcement, algorithmic updating, exposure diversity, cognitive-affective processes, and problematic engagement. Together, these strands support a platform-sensitive account of how repeated engagement may alter subsequent exposure and how the updated exposure environment may, in turn, shape later cognitive-affective responses and behavior. I-PACE remains a useful background account of how predisposing characteristics, affective and cognitive responses, cue-reactivity, executive functions, decision processes, and habit formation may contribute to problematic digital behavior (Brand et al., 2019). RNS overlaps with some of this vocabulary, but it addresses a different analytical problem: how user behavior changes the subsequently encountered feed and how that updated exposure environment may, in turn, alter attention, affect, and later behavior. The distinctive focus of RNS is the informational environment as an endogenous and measurable state. A user's behavior at time t changes the feed encountered at time t+1; that feed may alter attention, affect, and behavior at time t+2. After controlling for baseline vulnerability and total use, within-person feed convergence should therefore predict later automaticity, monitoring errors, or impairment, whereas exogenous diversity injection should weaken the proposed pathway. These predictions define the framework's distinctive empirical contribution and provide criteria for evaluating its explanatory value.

**Figure 1:**
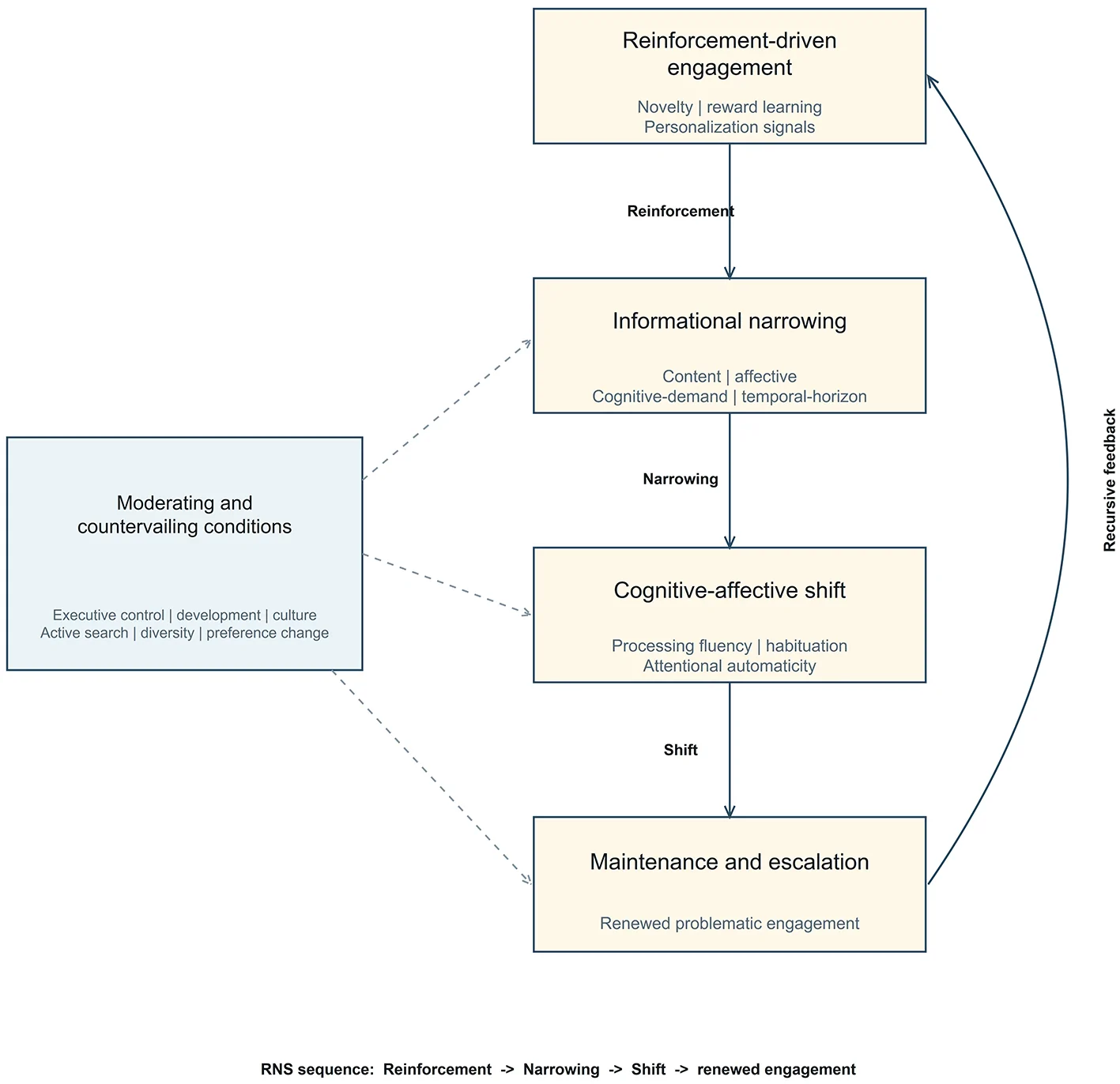
The recursive Reinforcement–Narrowing–Shift framework (RNS) for PSVU. This figure summarizes the recursive components and processes proposed in the RNS framework: reinforcement-driven engagement, informational narrowing, cognitive-affective shift, and maintenance or escalation through renewed problematic engagement. The feedback pathway represents re-entry into earlier reinforcement processes. Individual and contextual factors may moderate the strength, speed, and interruptibility of these relations.

### 3.1 Conceptual distinctions

Before presenting the recursive process in detail, it is necessary to distinguish four related concepts: filter bubbles, echo chambers, information cocoons, and informational narrowing (Table 1). These terms are often used interchangeably, but they differ in mechanism and level of analysis. Clarifying these distinctions helps specify why the present framework focuses on narrowing as a dynamic process rather than merely on homogeneous exposure as an outcome. Filter bubbles refer to algorithmic personalization that selectively presents content based on user characteristics and behavior, potentially limiting exposure to diverse information or viewpoints (Pariser, 2011). This is primarily a technological mechanism operating at the platform level. Echo chambers describe social network structures in which individuals are predominantly exposed to attitude-confirming information through homophilous connections and selective sharing (Sunstein, 2017). Echo chambers are therefore fundamentally social phenomena, although algorithmic systems may amplify them. Information cocoons, in contrast, refer to the user-level condition of being situated within an increasingly narrow informational environment. This condition may arise from algorithmic curation, social selection, self-selection, or their interaction (Zuiderveen Borgesius et al., 2016; Zhou, 2025; Hou et al., 2023). The concept captures the user's situated exposure environment and the behavioral consequences of narrowed informational access. In this framework, informational narrowing refers to the dynamic process through which informational diversity progressively contracts across multiple dimensions, whereas an information cocoon refers to the resulting user-level condition. This distinction matters because the framework explains PSVU through the process by which narrowed exposure becomes reinforced and difficult to interrupt.

**Table 1:** Conceptual distinctions: filter bubbles, echo chambers, information cocoons, and informational narrowing.

Concept	Definition	Primary mechanism	Level of analysis	Role in RNS
Filter bubble	Algorithmic selection that can reduce exposure to dissimilar information	Algorithmic curation	Platform/exposure	One possible technological route to narrowing, but not an inevitable outcome of personalization
Echo chamber	A network structure in which socially shared information repeatedly confirms group-consistent views	Homophily and selective sharing	Network	A socially produced concentration that may interact with algorithmic curation
Information cocoon	A user-level state of persistently restricted informational exposure	Self-selection, social selection, and/or algorithms	Individual exposure state	A possible condition resulting from sustained narrowing
Content-level narrowing	Declining thematic, source, format, or viewpoint diversity in the feed	Engagement-weighted selection under weak diversity constraints	Exposure	Directly measurable environmental component
Affective narrowing	Reduced diversity of emotional tone or repeated concentration on a limited set of high-engagement affective cues	Affective engagement optimization	Exposure	Directly measurable environmental component
Cognitive-demand narrowing	A shift toward content requiring less sustained integration, elaboration, or critical evaluation	Selection for rapid comprehension and response	Content-experience interface	Hypothesized experiential component; distinct from general cognitive decline
Temporal-horizon narrowing	Compression of attention and choice into short reward intervals with fewer reflective stopping points	Rapid sequencing and low-friction continuation	Use-episode structure	Experience-level structural component; distinct from disengagement failure and other behavioral outcomes

This table distinguishes related concepts that are often conflated in research on digital media environments. Filter bubbles emphasize algorithmic mechanisms, echo chambers emphasize social network structures, information cocoons describe the user-level condition of narrowed exposure, and informational narrowing refers to the dynamic process through which such narrowing develops.

### 3.2 The co-production process

Informational narrowing can emerge through recursive co-production between user behavior and algorithmic curation (Gillespie, 2014; Kitchin, 2017), but personalization does not necessarily cause narrowing. Recommendation systems may also introduce novelty, serendipity, and diversity. RNS therefore defines narrowing as a conditional within-person decline in exposure or experiential breadth, not as a synonym for personalization or a universally homogeneous feed. Co-production involves behavioral signal accumulation, algorithmic updating, and user adaptation. Watch time, viewing transitions, and explicit interactions provide temporally fine-grained behavioral signals (Bhandari and Bimo, 2022). Engagement-weighted systems may exploit stable signals by presenting content likely to elicit further interaction, while users adapt their expectations and actions to the changing stream. Engagement is not equivalent to welfare or stable preference: immediate choices may conflict with reflective goals, and optimization can move content in welfare-improving or welfare-reducing directions (Kleinberg et al., 2024). Field evidence that algorithmic matching changes interaction patterns further supports treating exposure as co-produced rather than merely selected by users (López Vargas et al., 2022). The loop is recursive rather than a one-time calibration. Yet its direction depends on platform objectives and user behavior. Narrowing is most plausible when engagement optimization is predominantly exploitative, user signals are repetitive, diversity constraints are weak, and consumption is passive and low-friction. Stable personalization without progressive contraction is plausible when preferences and ranking remain stable. Diversification is plausible when systems retain exploration, novelty, serendipity, or coverage objectives; when users actively search and follow heterogeneous sources; when preferences change; or when recommendation histories are reset. External events, social ties, and offline commitments can also interrupt the coupling. These boundary conditions make narrowing falsifiable and distinguish it from ordinary preference-based personalization.

#### 3.2.1 Boundary conditions and countervailing dynamics

The relevant empirical question is therefore not whether personalization exists, but whether exposure diversity declines within a person over time and under which ranking and use conditions. A feed can become more relevant without becoming narrower, and a narrow feed can reflect deliberate specialization rather than maladaptive loss of choice. In RNS, narrowing becomes theoretically important when it is persistent, not readily apparent to users, and difficult to reverse. Its consequences for behavioral flexibility and functional impairment should be examined separately. Direct platform evidence is beginning to clarify these boundary conditions. A one-year observational study of new users on a large short-video platform found increasing homogeneity in consumed content, but also showed that more accurate personalization did not necessarily produce more severe information cocoons and that narrow user preferences were important (Li et al., 2022). This pattern supports the RNS emphasis on user–algorithm co-production while cautioning against attributing narrowing to the algorithm alone.

### 3.3 Four dimensions of narrowing

Informational narrowing in SFV environments is multidimensional. To avoid mixing environmental properties with psychological outcomes, we distinguish two exposure-level dimensions—content-level and affective narrowing—from two experience-level dimensions—cognitive-demand and temporal-horizon narrowing. The latter describe the structure of content and use episodes, not generalized cognitive decline. Content-level narrowing refers to a within-person decline in thematic, source, format, or viewpoint diversity. It should be measured relative to an individual baseline and relevant platform opportunities, because concentrated exposure may reflect deliberate expertise or temporary goals rather than maladaptation. Topic entropy, source diversity, semantic distance, novelty, and exposure coverage are possible indicators. Affective narrowing refers to declining diversity in emotional tone or repeated concentration on a limited set of high-engagement affective cues, such as outrage, reassurance, amusement, or anxiety. It is not equivalent to greater emotional intensity. Valence and arousal distributions, emotion-category entropy, and within-session affective transitions can distinguish concentration from escalation. Direct causal evidence in SFV feeds remains limited. Cognitive-demand narrowing refers to a shift in consumed content toward lower requirements for sustained integration, elaboration, source comparison, or critical evaluation. This is a property of the content-experience interface, not evidence that users' general cognitive ability has deteriorated. It can be indexed through linguistic and semantic complexity, argument structure, information density, required viewing continuity, and behavioral comprehension measures. Temporal-horizon narrowing refers to a compression of the use episode into shorter reward intervals, denser transitions, and fewer opportunities for reflective choice. It is therefore a property of episode structure rather than a behavioral symptom of problematic use. Potential indicators include the spacing of explicit choice or stopping points, transition density, and the temporal distribution of successive content exposures. Stopping failures, intended–actual duration discrepancies, and time-estimation errors are treated instead as downstream outcomes that temporal-horizon narrowing may help explain. Short content is not inherently harmful; the proposed mechanism concerns repeated rapid sequencing combined with reduced opportunities for reflection. The four dimensions may covary but should be tested separately. Content and affective concentration may increase predictability; lower cognitive demands may facilitate rapid comprehension; and compressed temporal structure may favor immediate continuation. Countervailing dynamics are equally possible: unfamiliar content may raise cognitive load, diverse exposure may restore exploration, and fatigue or saturation may promote disengagement. RNS therefore predicts conditional relations among dimensions rather than a single inevitable contraction.

### 3.4 Three bridging mechanisms

The proposed transition from reinforced engagement to cognitive-affective shift involves three bridging mechanisms: processing fluency and metacognitive erosion, affective habituation and escalation, and attentional capture and automaticity. These are analytically distinct hypotheses. Evidence from adjacent domains establishes their plausibility but does not demonstrate that SFV narrowing causes each mechanism.

#### 3.4.1 Processing fluency and metacognitive erosion

Processing fluency is the subjective ease with which information is processed. Predictable, schema-consistent feeds may increase fluency and make continuation feel effortless (Alter and Oppenheimer, 2009). Cognitive load theory addresses a different process: working-memory demands created by complexity, rapid switching, or competing information (Sweller, 1988). A homogeneous and predictable feed may be fluent and low in perceived load, whereas a rapidly changing heterogeneous feed may be disfluent and cognitively demanding. RNS prioritizes fluency when explaining reduced monitoring in a convergent feed, but treats cognitive load as a complementary or competing explanation that must be manipulated independently. Direct SFV evidence remains limited. Experiments should independently manipulate content predictability and switching rate, then measure perceived fluency, cognitive load, recall, time estimation, source evaluation, and stopping behavior. Evidence for a narrowing pathway would require changes in feed composition to precede monitoring errors, rather than inferring metacognitive erosion from heavy use alone.

#### 3.4.2 Affective habituation and escalation

Repeated exposure to affectively arousing content can produce habituation, defined as a decreased emotional response to repeated or familiar stimuli (Thompson and Spencer, 1966). As users adapt to the affective intensity of a narrowed content stream, previously engaging content may produce weaker emotional responses. This may create pressure for affective escalation, whereby users become more responsive to more intense, novel, or emotionally charged content in order to achieve comparable levels of stimulation or relief (Zillmann, 1988). Recommendation systems may facilitate this process by continuously testing content variants and prioritizing those that restore or sustain engagement (Bhandari and Bimo, 2022). For example, a user initially engaging with mildly humorous videos may gradually receive more exaggerated or emotionally intense humor, while exposure to unrelated content domains decreases. In this way, users may experience increasing affective intensity within a progressively restricted content domain. This dynamic is conceptually analogous to tolerance-like processes described in addiction research, although the analogy should be interpreted cautiously. In the context of PSVU, the relevant process is not physiological tolerance but a possible shift in affective thresholds and stimulation preferences. The combination of affective escalation and informational narrowing may help explain why some users continue to engage even when ordinary content becomes less satisfying. Although direct SFV-specific evidence remains limited, related research on mood management, media violence, and algorithmic amplification of emotionally salient content provides a theoretical basis for this proposition (Zillmann, 1988; Ludwig et al., 2023; Habib and Nithyanand, 2025; see Table 2). Future longitudinal studies should track both content characteristics and users' affective responses over time to examine whether affective habituation predicts increased preference for more stimulating content.

**Table 2:** Testable propositions derived from the reinforcement–narrowing–shift framework.

Proposition	Statement	Boundary condition or theoretical basis	Recommended module and minimum measures
P1	Cue-triggered SFV use predicts larger gaps between intended and actual disengagement.	Automaticity and cue-reactivity	Module A: app-opening EMA plus passive logs of intended and actual session length.
P2	Repeated engagement predicts declining within-person exposure diversity when exploitation is emphasized over exploration.	Recommendation objective and exploration-exploitation balance	Module A: repeated feed donation plus topic, source, format, and semantic-diversity metrics.
P3	Diversity-aware or serendipity-oriented recommendation weakens or reverses the association between repeated engagement and exposure narrowing.	Algorithmic boundary condition	Module B: randomized diversity or serendipity injection with the same exposure-diversity metrics.
P4	Exposure narrowing mediates the association between user vulnerability and later PSVU beyond total use duration.	I-PACE plus endogenous environmental mediation	Module A: at least three waves of feed metrics, vulnerability measures, and validated PSVU indicators.
P5	Predictable, schema-consistent feeds increase processing fluency and reduce monitoring accuracy, whereas rapid heterogeneous feeds may instead increase cognitive load.	Fluency and cognitive-load accounts	Module B: factorial manipulation of predictability and switching rate; assess fluency, load, recall, and monitoring.
P6	Affective habituation predicts subsequent selection of more intense content within a restricted affective domain.	Habituation and incentive processes	Module B: repeated affective exposure with valence, arousal, habituation, and subsequent-choice measures.
P7	Exposure narrowing strengthens cue-induced craving and automatic engagement, particularly among users with weaker executive control.	Associative learning and executive-control moderation	Module B: cue-reactivity protocol with craving plus stop-signal or working-memory performance.
P8	Feed convergence predicts later attentional inflexibility or time-estimation error after controlling for baseline vulnerability and screen time.	RNS-specific incremental prediction	Module A: baseline and follow-up attention-flexibility or time-estimation tasks linked to feed convergence.
P9	Cultural, institutional, and platform conditions moderate both the rate of narrowing and the probability of spontaneous or supported exit.	Contextual moderation and natural exit pathways	Module C: apply the same Module A core measures across platforms or cultures and record exit events.

The modules are reusable study templates rather than nine separate methodological requirements. Module A is a repeated-measures digital-trace panel; Module B is a controlled feed experiment; Module C is a comparative extension of the same core protocol across platforms or contexts.

#### 3.4.3 Attentional capture and automaticity

As SFV use becomes repeatedly paired with situational and emotional cues, platform-related cues may increasingly capture attention and trigger automatic engagement. Such cues may include boredom, waiting, fatigue, loneliness, stress, bedtime routines, or the mere availability of the app on a mobile device. Through repeated pairing, these cues can become associated with SFV use and develop into automatic stimulus-response patterns that bypass deliberate decision-making (Wood and Rünger, 2016). Automaticity may manifest in several ways. Users may open the app without clear intention, continue scrolling despite prior plans to stop, or find their attention repeatedly drawn to the platform during study, work, or interpersonal interaction. Evidence from problematic social media and smartphone use suggests that platform-related cues can be associated with heightened cue-reactivity and altered reward or habit-related processing, although SFV-specific neurocognitive evidence remains emerging (He et al., 2017; Wadsley and Ihssen, 2023; Wolf et al., 2025). Informational narrowing may strengthen automaticity by increasing predictability and reducing the cognitive effort required for engagement. When content is familiar, fluent, and emotionally relevant, users may need less deliberative control to continue viewing. Over time, engagement may shift from goal-directed choice toward more automatic response patterns (Dickinson, 1985). Thus, algorithmic narrowing may shape not only what users see but also how they engage with the platform. These three bridging mechanisms are mutually reinforcing. Processing fluency may reduce metacognitive awareness of both habituation and automaticity. Affective escalation may strengthen cue-based associations by increasing the motivational salience of platform use. Automaticity may further reduce opportunities for reflective evaluation. Together, these mechanisms help explain why narrowed exposure may produce changes that are not easily addressed by reducing use duration alone.

### 3.5 Three-stage recursive model

The proposed framework conceptualizes PSVU as developing through three analytically distinct but potentially overlapping stages: initiation and reinforcement, consolidation through narrowing, and maintenance and escalation. These stages are analytical distinctions rather than diagnostic categories; in practice, they may overlap, recur, or stabilize differently across users and contexts. Consider a hypothetical user who initially opens an SFV app to find cooking tutorials. Early exploration produces a broad mix of recipes, travel, humor, and news. Repeated completion and re watching of visually intense late-night cooking clips gives the system increasingly consistent signals. If the ranking system emphasizes exploitation and the user mainly consumes passively, the feed may converge on highly stimulating food content delivered in rapid succession. Bedtime opening becomes cue-triggered, perceived session length becomes inaccurate, and the app is increasingly used to manage fatigue or stress. The cycle is not inevitable: active searches for other topics, a diversity-aware feed, content saturation, a change in work schedule, or deliberate stopping cues could stabilize, reverse, or interrupt it.

#### 3.5.1 Stage 1: initiation and reinforcement

Initial engagement with SFV platforms is often characterized by exploration, novelty-seeking, and relatively broad content exposure. Users may encounter diverse content while recommendation systems collect behavioral data to calibrate future recommendations. During this phase, engagement is supported by novelty, curiosity, social cues, and intermittent reinforcement generated by variable and unpredictable content rewards (Ferster and Skinner, 1957). Platform affordances, including infinite scroll, auto play, full-screen presentation, and low-friction transitions between videos, can facilitate extended viewing even before personalization becomes highly refined (Zhao and Wagner, 2023). Users experience frequent “wins,” such as funny, useful, emotionally relevant, or socially meaningful videos, interspersed with less engaging content. This variable reinforcement structure encourages continued use and provides the behavioral data on which personalization depends. Individual vulnerability factors shape this initial stage. Users experiencing boredom, stress, loneliness, negative affect, or fear of missing out may find SFV platforms especially reinforcing as tools for distraction or mood regulation (Elhai et al., 2020; Chen et al., 2024). Users with higher impulsivity or lower self-control may be more susceptible to immediate gratification and low-friction continuation (Du et al., 2019). Importantly, these individual characteristics do not operate in isolation; they interact with platform affordances to shape the likelihood that routine use becomes repetitive and cue-triggered.

#### 3.5.2 Stage 2: consolidation through narrowing

As behavioral signals accumulate, recommendation systems become increasingly personalized, initiating the multidimensional narrowing process described in Section 3.3. This stage is characterized by stronger coupling between user behavior and algorithmic output. Content becomes more predictable, affectively targeted, cognitively fluent, and temporally compressed. Users may experience this stage positively because the feed appears increasingly relevant and aligned with their interests (Klug et al., 2021). However, the perceived improvement in personalization may coincide with less visible cognitive–affective shifts. Processing fluency may increase while metacognitive monitoring weakens. Affective habituation may begin as users adapt to the emotional tone of their feed. Time distortion may become more pronounced as rapid sequential viewing reduces opportunities for reflective disengagement. The transition from Stage 1 to Stage 2 is marked by a shift in the dominant driver of engagement. Whereas Stage 1 is primarily supported by novelty and exploration, Stage 2 is increasingly supported by habit, predictability, and automatic response. Users may begin to experience difficulty disengaging even when the content is no longer especially novel or rewarding, suggesting that continued use has become partially decoupled from immediate gratification and increasingly governed by learned cue-response patterns.

#### 3.5.3 Stage 3: maintenance and escalation

In Stage 3, problematic patterns may become consolidated when narrowed exposure, automatic engagement, and cognitive-affective responses continue to reinforce one another. This stage is defined by persistence and reduced flexibility, not by a diagnostic threshold. The strongest test would show that earlier feed convergence predicts later control difficulty or impairment beyond prior symptoms and use duration. Several features distinguish this stage. First, users may experience persistent difficulty controlling use despite intentions to reduce or stop, indicating reduced reflective control and increased automaticity (Griffiths, 2005; Ciudad-Fernández et al., 2024). Second, affective escalation may become more evident as users seek more stimulating, emotionally intense, or novelty-rich content to achieve comparable affective effects. Third, functional impairment may emerge as SFV use interferes with sleep, study, work, interpersonal relationships, physical activity, or psychological wellbeing. Importantly, Stage 3 is maintained not only by individual habit strength but also by environmental entrenchment. Users may become embedded in a narrowed content ecosystem that continuously reinforces familiar emotional and attentional patterns while providing limited exposure to alternative information, activities, or regulatory strategies that might disrupt the cycle. This environmental component distinguishes algorithm-driven problematic use from accounts that focus solely on individual self-control. Stage 3 is neither inevitable nor terminal. Natural exit pathways may arise through content fatigue, changing life circumstances, or migration to other platforms. Deliberate self-regulation, social support, educational interventions, recommendation resets, or platform design changes may also weaken coupling. These pathways should be modeled as competing transitions rather than treated as unexplained attrition.

### 3.6 Testable propositions

The RNS framework generates nine falsifiable propositions (Table 2). They are intended as a phased research agenda rather than requirements for a single omnibus study. To improve feasibility, the propositions are organized into three reusable modules: a repeated-measures digital-trace panel, a controlled feed experiment, and a comparative extension that applies the same core measures across platforms or contexts. Several propositions remain directly rejectable: repeated use may not reduce diversity, diversity injection may not weaken automaticity, or feed convergence may add no predictive value beyond individual vulnerability and total use. Module A, the repeated-measures digital-trace panel, combines brief app-opening EMA, passive session logs, repeated feed sampling or consent-based data donation, and validated PSVU and executive-control measures; it can address P1, P2, P4, and P8. Module B, the controlled feed experiment, manipulates diversity, predictability, switching rate, or cue exposure while reusing common measures of fluency, affect, craving, monitoring, and inhibitory control; it can address P3, P5, P6, and P7. Module C applies either protocol across platforms, cultures, or institutions and records spontaneous and supported exits to address P9. These modules can be implemented separately and later integrated through coordinated or multisite studies. Data minimization, transparent consent, secure processing, and sensitivity analyses remain necessary for donated or tracked data (Boeschoten et al., 2022; Ohme et al., 2024). The propositions are a research agenda rather than confirmation of the complete cycle. Existing studies provide component-level evidence concerning flow, affect regulation, attentional control, time perception, sleep, academic functioning, and neurocognitive correlates, but many are cross-sectional, self-reported, geographically concentrated, or unable to observe recommendation output. Supplementary Table S1 therefore maps evidence to components and limitations rather than labeling every study as support for the integrated framework.

## 4 Cultural and institutional moderators: why the loop varies across contexts

The recursive framework developed in Section 3 describes mechanisms through which PSVU may become self-maintaining through the interaction of reinforcement, informational narrowing, and cognitive–affective shifts. However, these mechanisms do not operate in a sociocultural vacuum. Cultural norms and institutional environments may shape the intensity, visibility, and interruptibility of the RNS cycle. In the present framework, cultural and institutional factors are conceptualized as contextual moderators rather than as separate causal mechanisms. They may amplify or attenuate the processes described above by shaping motivational entry points, interpretations of narrowing, access to alternative regulatory strategies, and the availability of structural exit pathways. Because direct evidence on cultural and institutional moderation of PSVU remains limited, this section draws cautiously on related research on social media use, smartphone overuse, problematic Internet use, and general digital media engagement. The discussion should therefore be read as a theoretically grounded agenda for future cross-cultural and cross-institutional research rather than as conclusive evidence.

### 4.1 Cultural moderation of motivational entry and narrowing interpretation

Cultural contexts may shape both why users enter the problematic use cycle and how they interpret the narrowing of their informational environment. Cross-cultural psychology has long distinguished between cultural orientations emphasizing personal autonomy, self-expression, and independence, and those emphasizing social harmony, relational obligation, and interdependence (Markus and Kitayama, 1991; Triandis, 1995). These orientations can influence media-use motivations, social comparison patterns, emotion regulation norms, and vulnerability to problematic digital engagement (Kim et al., 2011; Kircaburun et al., 2020; Yang, 2022). In contexts characterized by more interdependent or collectivistic orientations, SFV platforms may be especially salient as spaces for social connection, peer alignment, identity validation, and participation in shared trends. Users may be motivated to consume and share content that signals belonging, maintains social ties, or aligns with group norms. In such contexts, informational narrowing toward group-congruent, socially validated, or trend-based content may be experienced less as a restriction than as an extension of social participation, thereby strengthening affective pathways related to social approval, fear of missing out, and interpersonal validation (Kim et al., 2011; Luo et al., 2024). By contrast, in contexts characterized by more independent or individualistic orientations, SFV use may be more closely linked to self-presentation, identity exploration, entertainment, and personalized choice. Users may be more likely to interpret algorithmic narrowing as a constraint on autonomy or informational diversity and may express stronger preferences for transparency, user control, and the ability to modify recommendation systems, as suggested by research on algorithmic awareness conducted largely in Western platform contexts (Eslami et al., 2015; Rader and Gray, 2015; Harambam et al., 2018). However, such awareness does not necessarily lead to behavioral diversification, because even users who recognize personalization may continue to engage with narrowed feeds when they are convenient, emotionally rewarding, and socially validated.

Cultural norms regarding emotion expression may also moderate the affective pathways proposed in Section 3.4. In contexts where emotional restraint is normative, users may rely on SFV platforms for private mood regulation while expressing fewer overt signs of emotional dependence. In contexts where emotional expressiveness is more socially accepted, affectively charged engagement may become more visible through commenting, sharing, or trend participation. These behaviors may elicit stronger interpersonal feedback from other users, thereby strengthening social-validation pathways (Mesquita and Frijda, 1992; Tsai et al., 2006). These differences may alter the relative salience of affective habituation, social validation, and cue-triggered engagement. These cultural patterns should not be interpreted deterministically. Variation within cultural groups is often substantial, and globalization, migration, acculturation, and platform-specific youth cultures complicate simple cultural categorizations (Chiu and Hong, 2006; Valkenburg and Peter, 2011). Moreover, the same user may display different motivational patterns across platforms and life contexts. The implication for the present framework is therefore not that culture directly determines PSVU, but that cultural context may moderate which bridging mechanisms—processing fluency and metacognitive erosion, affective habituation and escalation, or attentional capture and automaticity—become most salient in driving the transition from informational narrowing to cognitive–affective shift.

### 4.2 Institutional moderation of cycle intensity and interruptibility

Institutional environments may shape the intensity of the RNS cycle and the availability of exit pathways. These environments include regulatory frameworks, platform governance structures, educational systems, family and school norms, and public health infrastructure. Their effects are likely to operate across multiple levels and may interact with cultural values.

#### 4.2.1 Regulatory frameworks and platform governance

Regulatory frameworks can influence whether platforms provide transparency, user control, diversity safeguards, and friction against problematic engagement. Measures may include non-profiled feed options, meaningful recommendation controls, age-appropriate defaults, independent researcher access, and auditable risk-management processes. The European Union's Digital Services Act and Chinese youth-mode policies illustrate different institutional approaches, although implementation and effectiveness require empirical evaluation (European Commission, 2022; State Council of the People's Republic of China, 2023; Solomatina, 2025). In contrast, institutional contexts with limited oversight of algorithmic curation or persuasive design may provide fewer structural protections against engagement-maximizing mechanisms. Where platforms face little regulatory or civil-society pressure to modify engagement-maximizing design features, low-friction continuation and algorithmic convergence may operate more intensively (Zuboff, 2019; Susser et al., 2019). These institutional differences may influence both the baseline strength of reinforcement and the likelihood that users encounter meaningful interruption points. Platform governance can also incorporate science-based detection and stepped intervention. Gambling regulation provides adjacent examples of algorithmic systems designed to identify risk from behavioral traces, while systematic reviews emphasize personalized feedback, pre-commitment, and self-exclusion alongside modest effects and limited evidence for durability (Hayer and Kalke, 2022). A behavior-evaluation mismatch—continued or increasing use despite a negative reflective evaluation—may offer a minimally invasive risk signal (Runge, 2025). Transfer to SFV contexts, however, requires direct validation. Engagement traces alone should not be used to infer disorder. Any risk-detection system should use informed consent, transparent decision rules, and independent oversight to minimize misclassification, privacy harms, and stigma.

#### 4.2.2 Educational systems, family norms, and media literacy

Educational systems can moderate the transition from informational narrowing to cognitive-affective shift by shaping users' algorithmic awareness, critical evaluation skills, and metacognitive monitoring. Media and algorithmic literacy programs may help users understand how recommendation systems shape exposure, how engagement signals influence future feeds, and how personalization can narrow informational diversity (Hobbs, 2010; Livingstone et al., 2017; Buckingham, 2019). However, awareness alone is unlikely to be sufficient. Users may understand that algorithms personalize content but still continue to engage with narrowed feeds because they are fluent, emotionally satisfying, and low effort (Harambam et al., 2018). Educational interventions are therefore likely to be more effective when they combine algorithmic literacy with practical skills, such as feed auditing, content diversification exercises, reflective pausing, goal-setting, and emotion-regulation alternatives. Family and peer environments also function as institutional and social moderators, especially for adolescents. Contexts that normalize high-frequency platform use, bedtime scrolling, or constant peer comparison may accelerate reinforcement and narrowing. Conversely, environments that encourage balanced media routines, critical discussion of online content, and exposure to diverse offline activities may slow the development of problematic patterns (Valkenburg and Piotrowski, 2017; Beyens et al., 2020). The effectiveness of parental or school-based mediation may vary by cultural context. In some settings, explicit rules and monitoring may be seen as normative and protective; in others, the same strategies may be experienced as autonomy-threatening and may generate resistance.

#### 4.2.3 Public health infrastructure and mental health support

Public health infrastructure provides another potential moderator of the cycle's interruptibility. Because PSVU often intersects with stress, loneliness, anxiety, boredom, sleep problems, and emotion-regulation difficulties, accessible mental health support may provide alternative pathways for coping before SFV use becomes the dominant regulatory strategy. In contexts where psychological services are available, affordable, and socially accepted, users may have more opportunities to interrupt the transition from affect-regulation use to consolidated problematic engagement (Patel et al., 2018). By contrast, when mental health resources are scarce, stigmatized, or difficult to access, SFV platforms may become more attractive as immediate and private sources of distraction, mood repair, and social contact. This does not mean that limited mental health infrastructure directly causes PSVU. Rather, it may reduce the availability of alternative regulatory resources, thereby increasing reliance on platform-based affect regulation and making the recursive cycle harder to interrupt.

### 4.3 Implications for research and intervention

The cultural and institutional moderators discussed above have two major implications. First, they highlight the need for cross-cultural and cross-institutional research on PSVU. The mechanisms described in Section 3 may be broadly applicable, but their relative salience, thresholds for stage transitions, and interruptibility are likely to vary across contexts. Current research on SFV use remains concentrated in particular populations and institutional settings, especially student samples and East Asian contexts, which limits generalizability. Future research should use comparative designs to test whether the RNS cycle operates similarly across platforms, age groups, cultures, and regulatory environments (Henrich et al., 2010; Montag et al., 2021).

Second, these moderators point to the need for multilevel and culturally calibrated interventions. Individual-level strategies may be insufficient when platform environments continue to prioritize engagement maximization, whereas platform-level changes may have limited effects if users lack the literacy, motivation, or social support needed to use them effectively. Effective intervention therefore requires aligned actions across levels: platforms can provide safeguards that facilitate disengagement, institutions can provide literacy and support, and users can apply practical self-regulation strategies. Cultural calibration is also important. In contexts where social validation and group belonging are central motives, interventions may benefit from peer-based support and collective media-balance practices. In contexts where autonomy and personal choice are more salient, interventions may be more effective when framed around transparency, self-direction, and user control. These implications are developed more fully in the next section.

## 5 Intervention implications: a mechanism-matched multilevel framework

The intervention framework proposed here is theoretically grounded in the mechanisms developed in Sections 3 and 4. Because direct evidence for SFV-specific interventions remains limited, this section draws on related research on problematic smartphone use, problematic social media use, digital self-control tools, recommender-system design, and media literacy. The aim is not to prescribe a fixed intervention package, but to identify where different strategies may interrupt the recursive RNS cycle.

### 5.1 Mapping interventions to cycle linkages

The RNS framework suggests that effective intervention requires more than reducing screen time or encouraging individual willpower. If PSVU is maintained through linkages among reinforcement, informational narrowing, cognitive–affective shifts, and renewed engagement, then interventions should target the mechanisms sustaining these linkages. Accordingly, we propose a mechanism-matched framework with three primary intervention levels plus institutional coordination. Platform-level strategies target the transition from reinforcement-driven engagement to informational narrowing; educational strategies target the transition from informational narrowing to cognitive-affective shift; and user-level strategies target the transition from cognitive-affective shift to renewed problematic engagement. Figure 2 and Table 3 represent these primary intervention targets consistently.

**Figure 2:**
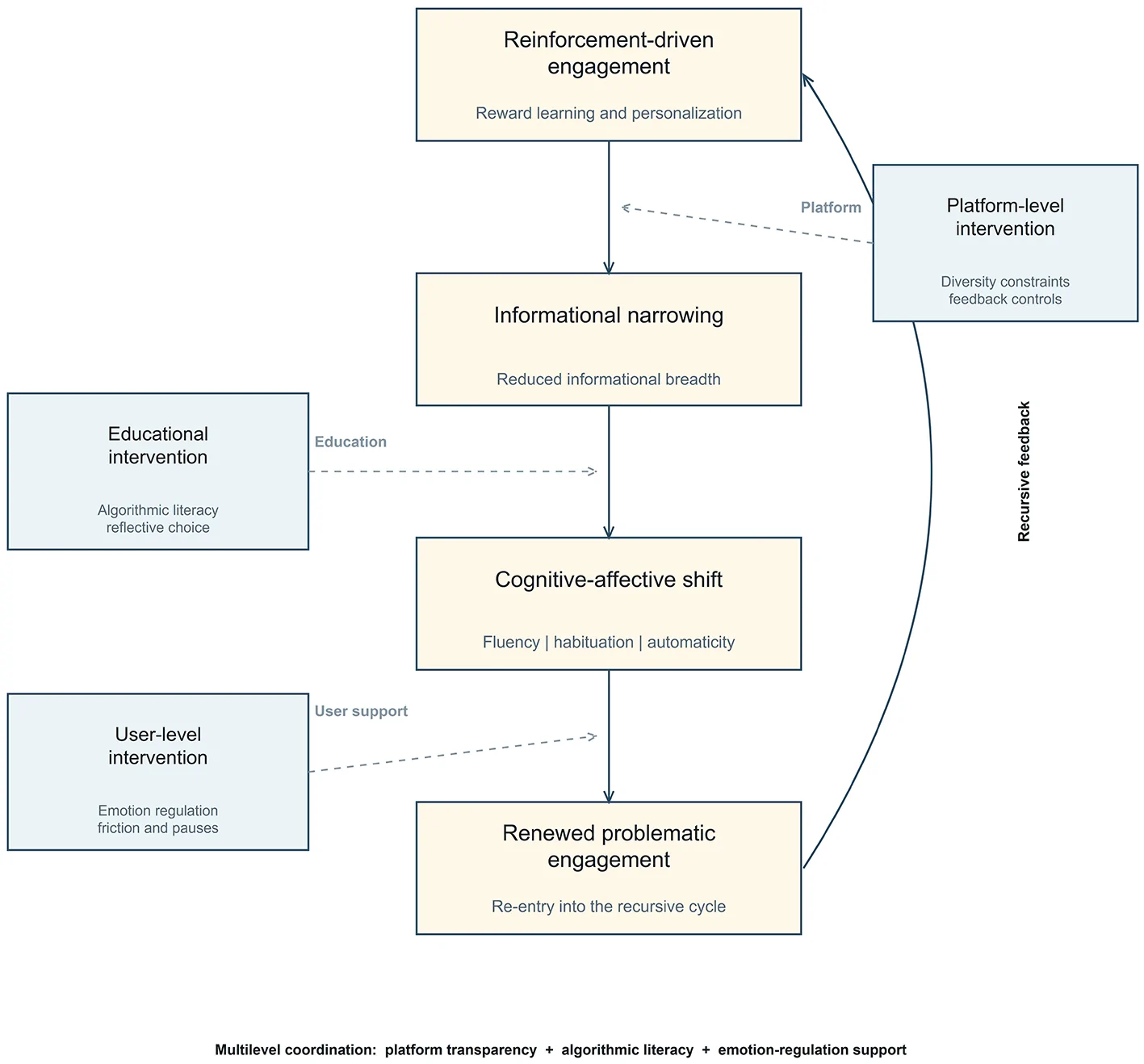
Mechanism-matched multilevel intervention framework.

**Table 3:** Mechanism-matched multilevel intervention framework.

Intervention level	Primary target represented in Figure 2	Core logic	Representative strategies	Evidence and safeguards
Platform-level	Reinforcement-driven engagement → informational narrowing	Reduce low-friction re-entry, engagement-only optimization, and rapid convergence	Autoplay/session limits; stopping prompts; diversity-aware ranking; recommendation reset; preference controls; risk detection using behavior-evaluation discrepancies	Adjacent-domain evidence; require consent, explainability, privacy protection, error monitoring, human review, and independent evaluation
Educational	Informational narrowing state and its transition toward cognitive-affective shift	Make curation and loss of diversity visible; strengthen metacognitive monitoring	Algorithmic literacy; feed audits; diversity reflection; source checking; cognitive-load and fluency awareness	Knowledge gains are more established than durable behavior change; avoid treating awareness as sufficient
User-level	Cognitive-affective shift → renewed engagement	Interrupt cue-triggered use and diversify emotion regulation	Implementation intentions; self-monitoring; emotion-regulation alternatives; environmental restructuring; planned breaks	Related-domain evidence is promising but persistence and individual burden remain concerns
Institutional/multilevel	Conditions shaping the entire cycle	Align platform accountability, user support, and scientific evaluation	Auditable risk systems; age-appropriate design; independent data access; education and mental-health support	Transfer from gambling/gaming must be tested in SFV settings; avoid clinical labeling from engagement data alone

These strategies should be understood as theoretically grounded directions for future SFV-specific intervention research rather than established treatments.

Figure 2 maps three primary intervention entry points onto the recursive cycle. Platform-level intervention targets the reinforcement-driven-engagement-to-informational-narrowing linkage, educational intervention targets the informational-narrowing-to-cognitive-affective-shift linkage, and user-level intervention targets the cognitive-affective-shift-to-renewed-problematic-engagement linkage. Because effects may propagate to adjacent components, the arrows denote primary rather than exclusive targets.

### 5.2 Platform-level intervention: weakening reinforcement before narrowing stabilizes

Platform-level interventions can weaken the transition from reinforcement-driven engagement to informational narrowing by reducing low-friction continuation, tempering engagement-only optimization, slowing algorithmic convergence, and introducing diversity constraints or reflective stopping points. These changes are intended to reduce the accumulation of repetitive signals and exposure convergence before narrowing stabilizes. Diversity-aware recommendation can incorporate novelty, serendipity, source coverage, and user-defined objectives alongside engagement metrics (Kaminskas and Bridge, 2017; Helberger et al., 2018). Friction tools include autoplay limits, session-end prompts, delayed continuation, and bedtime reminders. Science-based monitoring may combine clickstream indicators with brief reflective assessments to detect evaluation-use discrepancies (Runge, 2025), but should trigger supportive options rather than clinical labels. Any such system requires privacy protection, error monitoring, user appeal, transparent thresholds, and independent evaluation. Evidence from gambling and gaming can inform design but cannot be assumed to generalize to SFV (Hayer and Kalke, 2022).

### 5.3 Educational-level intervention: making narrowing visible before it becomes normalized

Educational interventions target the transition from informational narrowing to cognitive-affective shift. Their primary goal is to strengthen users' metacognitive capacity to recognize how algorithmic curation shapes exposure, attention, emotion, and behavior. This level is important because informational narrowing may be experienced as convenience, relevance, or entertainment rather than as a restriction. Algorithmic literacy programs can help users understand how behavioral signals, such as dwell time, completion rates, re watches, likes, comments, shares, and skips, shape future recommendations (Livingstone et al., 2017; Buckingham, 2019; Oeldorf-Hirsch and Neubaum, 2025). However, literacy should be paired with practical exercises. Feed auditing, information-cocoon awareness tasks, content-diversification exercises, and critical evaluation training may translate abstract knowledge into concrete metacognitive awareness. Because narrowed and fluent feeds may encourage heuristic processing, such training should also promote source checking, slower evaluation, and reduced reliance on popularity cues or emotional intensity (Pennycook and Rand, 2021). The main limitation is the awareness-behavior gap. Users may recognize narrowing but continue using personalized feeds because they remain rewarding, socially validated, and low effort (Harambam et al., 2018). Educational interventions should therefore be evaluated not only by knowledge gains but also by outcomes such as increased feed diversity, improved time-estimation accuracy, greater self-monitoring, reduced automatic checking, and greater willingness to use platform controls.

### 5.4 User-level intervention: restoring reflective interruption at moments of strain

User-level interventions target the final linkage in the recursive cycle: the transition from cognitive–affective shift to renewed engagement. They are especially relevant when SFV use has become cue-triggered, emotionally regulating, and difficult to stop. The goal is not to suppress all SFV use, but to restore flexibility in attention, emotion regulation, and behavioral choice. Promising strategies include implementation intentions, self-monitoring, emotion-regulation training, and attentional or executive-control supports. Implementation intentions link high-risk cues to alternative responses and may help users interrupt automatic app opening or bedtime scrolling (Gollwitzer and Sheeran, 2006). Self-monitoring can increase awareness of trigger patterns and discrepancies between intended and actual use duration. Emotion-regulation training may reduce reliance on SFVs as the dominant strategy for managing boredom, stress, loneliness, anxiety, or fatigue by strengthening alternatives such as reappraisal, problem-solving, mindfulness, relaxation, physical activity, social connection, and structured offline routines (Aldao et al., 2010). Attentional-control exercises, notification management, environmental restructuring, and planned breaks may further reduce cue-triggered engagement. However, user-level strategies should not place full responsibility on individuals. When platforms are designed to maximize low-friction engagement, self-control strategies may be difficult to sustain. User-level interventions are therefore likely to be most effective when supported by platform-level friction and educational-level awareness.

### 5.5 Why multilevel coordination is necessary

Single-level interventions are unlikely to fully disrupt the recursive RNS cycle because each level addresses only part of the mechanism. Platform changes may weaken reinforcement, educational programs may increase recognition of narrowing, and user-level strategies may strengthen self-regulation, but none of these alone addresses the full feedback process. Interventions should therefore be evaluated by whether they disrupt the mechanisms proposed in this framework, not only by whether they reduce screen time. Relevant outcomes include increased exposure diversity, improved disengagement success, reduced cue-triggered checking, more accurate time perception, stronger metacognitive monitoring, greater diversity of emotion-regulation strategies, and reduced functional impairment. By targeting the specific linkages that sustain the recursive cycle, coordinated multilevel interventions may offer a more precise approach to preventing and reducing problematic SFV use.

## 6 Limitations and future directions

Several limitations qualify the proposed RNS framework. Its causal ordering and full recursive structure remain untested, and most available evidence is cross-sectional, self-reported, or limited to isolated components. Reverse causality is plausible, and the framework should therefore be treated as a falsifiable research agenda rather than a validated diagnostic or causal model. A practical first step is a repeated-measures protocol linking passive use logs, feed sampling, brief EMA, and validated PSVU and executive-control measures, followed by targeted feed experiments rather than an attempt to test the complete cycle in one study. Measurement also remains underdeveloped. Future studies should distinguish feed composition, use-episode structure, and user outcomes. Content and affective narrowing can be estimated from donated feeds or screen recordings using diversity, novelty, and transition metrics. Cognitive-demand and temporal-horizon narrowing can be assessed using content complexity, sustained-attention requirements, transition density, and the spacing of reflective choice points. Stopping failures, intention–behavior discrepancies, time-estimation errors, and functional impairment should be measured separately as downstream outcomes. Data donation and screen tracking should be paired with self-report or laboratory measures while addressing consent, selection bias, missing content, and platform-specific data structures (Boeschoten et al., 2022; Ohme et al., 2024).

Narrowing and diversification should be studied symmetrically and across platforms, cultures, and institutional settings. Low diversity may reflect deliberate specialization rather than maladaptation, while exploration, active search, recommendation resets, content saturation, changing preferences, social support, and offline commitments may stabilize or interrupt the cycle. Comparative designs should identify which mechanisms generalize, which require contextual calibration, and which trajectories end without impairment. Intervention evidence remains preliminary. Direct SFV studies should compare platform friction, diversity-aware ranking, algorithmic literacy, and user-level regulation alone and in combination, using shared outcomes such as exposure diversity, disengagement success, time perception, metacognitive monitoring, functional impairment, adverse effects, and durability. Automated risk detection also requires calibration, privacy protection, transparent thresholds, and safeguards against labeling ordinary engagement as disorder. RNS will have added value only if changes in the exposure environment improve explanation or prediction beyond established individual-level factors.

## 7 Conclusion

This article proposed the RNS framework to explain how problematic short-form video use may emerge and persist through reciprocal relations among reinforcement, informational narrowing, cognitive-affective shifts, and renewed engagement. The model does not equate intensive use with pathology and does not assume that personalization necessarily narrows exposure. The central conditional claim is that engagement-dominant optimization, repetitive behavioral signals, weak diversity constraints, and passive low-friction consumption can produce exposure convergence that becomes increasingly difficult to recognize or interrupt. Exploration, serendipity, active search, changing preferences, natural fatigue, and life transitions can instead stabilize, diversify, or weaken the cycle. By treating the informational environment as an endogenous and measurable state, RNS offers a new conceptual synthesis derived from the structured integrative review. Future research should test whether feed convergence predicts later psychological and behavioral outcomes beyond vulnerability and screen time, whether diversity interventions disrupt the pathway, and when natural exits occur. Such tests will determine whether RNS provides explanatory value beyond its usefulness as an organizing framework.
