# Supplementary material for: Toward a recursive process model of problematic short-form video use: the Reinforcement–Narrowing–Shift framework
Source: Front Psychol. 2026 Oct 30;17:1871950. doi: 10.3389/fpsyg.2026.1871950 (PMC13760195; doi:10.3389/fpsyg.2026.1871950)
Supplement: Supplementary file 1 [file Supplementary_file_1.docx]

**Supplementary Table S1 Empirical Evidence Relevant to Components of the RNS Framework**

| **No.** | **Framework component** | **Study** | **Sample/method** | **Key finding** | **Evidence domain** | **Direction** | **Relevance and limitation** |
| --- | --- | --- | --- | --- | --- | --- | --- |
| 1 | Platform quality → flow → addiction | Qin et al., 2022 | Chinese adolescents aged 10–19; **N = 659**; questionnaire survey; Partial Least Squares analysis; S–O–R model and flow theory | System quality was strongly associated with flow, and flow was associated with TikTok addiction behavior. | Direct SFV | Component-consistent | Provides evidence relevant to platform quality, flow, and continued engagement. Cross-sectional self-report; temporal ordering and actual recommendation exposure were not observed. |
| 2 | Stress → flow → problematic use | Huang et al., 2022 | Middle-aged Chinese adults; **N = 194**; online cross-sectional survey; mediation analysis | Stress was associated with problematic SFV use through use duration and flow. | Direct SFV | Component-consistent | Relevant to affect-regulation and flow-related pathways into problematic use. Cross-sectional design does not establish causal direction. |
| 3 | Stress-coping pathway | Mu et al., 2022 | Chinese adolescents under 18 with SFV experience; **N = 242**; questionnaire survey; covariance-based SEM | School burnout and social phobia were associated with higher PSVU, which was linked to poorer psychosocial outcomes. | Direct SFV | Component-consistent | Provides evidence relevant to stress-coping pathways and downstream psychosocial correlates. Cross-sectional SEM cannot establish causal direction. |
| 4 | Socio-technical features → attachment → addiction | Zhang et al., 2019 | Short-form video app users; **N = 388** valid questionnaires; SEM | Social and platform factors were associated with attachment, which was associated with SFV app addiction. | Direct SFV | Component-consistent | Relevant to socio-technical and attachment-based reinforcement pathways. Cross-sectional questionnaire data; personalization and attachment were self-reported. |
| 5 | Excessive use across cultures | Yang, 2022 | Two parallel survey studies in China and the United States; Chinese Douyin users and U.S. TikTok users | Chinese and U.S. users differed in usage patterns, influencer preferences, and purchase-related responses. | Direct SFV | Mixed/contextual | Relevant to cultural variation in SFV use and platform experience. Cross-sectional self-report; platform, sampling, and cultural equivalence require caution. |
| 6 | Short-video addiction → academic procrastination | Xie et al., 2023 | Chinese undergraduates; **N = 1,047**; stratified random cluster sampling; cross-sectional survey; PROCESS Models 4 and 7 | PSVU predicted academic procrastination through attentional control; boredom proneness moderated the pathway. | Direct SFV | Component-consistent | Relevant to attentional-control and executive-regulation pathways. Cross-sectional moderated-mediation analysis; feed composition was not measured. |
| 7 | Short-video addiction → sleep quality | Jiang and Yoo, 2024 | Adolescents; **N = 1,629**; cross-sectional questionnaire survey; mediation analysis | PSVU was associated with poorer sleep quality, mediated by social anxiety. | Direct SFV | Component-consistent | Relevant to maintenance-stage functional impairment. Cross-sectional association; sleep, anxiety, and problematic use may influence one another. |
| 8 | Short-video addiction → academic anxiety/engagement | Li et al., 2024 | Chinese undergraduates; **N = 1,879**; questionnaire survey; structural equation modelling | SFV app addiction was associated with higher academic anxiety and lower academic engagement via mindfulness. | Direct SFV | Component-consistent | Relevant to academic impairment and reduced mindful awareness. Cross-sectional SEM; mindfulness is not a direct measure of metacognitive monitoring. |
| 9 | Short-video overuse → depression | Zhang D. et al., 2024 | College students; **N = 331**; two-wave follow-up with a 2-month interval; cross-lagged analysis | Earlier short-video overuse predicted later depression, but depression did not predict later overuse. | Direct SFV | Temporally supportive | Provides longitudinal evidence relevant to downstream affective consequences. Two waves improve temporal ordering but do not by themselves establish causality; feed exposure was not measured. |
| 10 | Time distortion | Yang et al., 2024 | **Study 1: N = 111** college students; three-phase survey with subjective and objective duration measures. **Study 2: N = 62** college students; randomized TikTok-versus-reading experiment | Short-form video use was associated with upward time distortion and overestimation of perceived duration. | Direct SFV | Experimentally supportive | Relevant to temporal-horizon narrowing and time-estimation processes. Effects were measured over short time periods and do not establish persistent temporal narrowing. |
| 11 | Risk decision-making | Zhang and Li, 2025 | College students; **N = 45** in the experiment (**SVA = 23; control = 22**); BART + fNIRS; 2 × 2 × 2 mixed experimental design | The high-SVA group showed greater risk taking and cue-related differences in prefrontal activation. | Direct SFV | Component-consistent | Provides neurobehavioral evidence relevant to cue sensitivity and altered risk decision-making. Small selected sample; group classification was based on extreme SVA scores, and ecological generalizability is limited. |
| 12 | Brain structure/function correlates | Gao et al., 2025 | College students; **N = 111** final sample; structural MRI and resting-state fMRI; neuroimaging analyses combined with transcriptomic/cellular mapping | SVA symptoms were associated with neuroanatomical and functional substrates; dispositional envy was behaviorally associated with SVA. | Direct SFV | Component-consistent | Relevant to neurocognitive correlates of SVA. Observational neuroimaging cannot determine whether neural differences precede or follow problematic use. |
| 13 | Personality → depression/anxiety → SVA | Zhang L. et al., 2024 | Chinese university students from seven universities; **N = 804**; cross-sectional survey; SPSS PROCESS chain-mediation analysis | Neuroticism and agreeableness were associated with SVA through depression and anxiety. | Direct SFV | Component-consistent | Relevant to vulnerability and affective pathways into problematic use. Cross-sectional mediation does not establish temporal or causal ordering. |
| 14 | Temporal focus and self-control | Liu et al., 2025 | University students; **N = 2,239**; questionnaire survey; variable-centered SEM/mediation and person-centered latent-profile analyses | Past/present focus was positively associated with SVA, while future focus showed a negative association; self-control mediated these relationships. | Direct SFV | Component-consistent | Relevant to temporal orientation and self-control mechanisms. Cross-sectional design limits causal interpretation; time focus may precede, accompany, or follow problematic use. |
| 15 | Attention and memory | Al-Leimon et al., 2025 | Jordanian university students across 25 higher-education institutions; **N = 1,029**; cross-sectional survey; mediation/moderation analyses | Attention partially mediated the association between SVA and memory function. | Direct SFV | Component-consistent | Relevant to attentional and cognitive correlates beyond Chinese samples. Cross-sectional self-report limits causal interpretation and may involve shared-method variance. |
| 16 | Flow, escapism, mood modification | Hong et al., 2025 | Chinese young adults; **N = 2,822**; questionnaire survey; network analysis | Flow, escapism, and mood modification were central nodes in the SVA network. | Direct SFV | Component-consistent | Provides direct SFV evidence relevant to reinforcement and affect-regulation mechanisms. Network centrality reflects statistical interconnection, not causal direction. |
| 17 | Binge-scrolling and loss of self-control | Park and Jung, 2024 | Exploratory sequential mixed-method study; qualitative and quantitative components; S–O–R framework | Infinite scrolling was associated with loss of self-control, regret, and reduced cognitive engagement. | Direct SFV | Component-consistent | Relevant to low-friction continuation and perceived loss of control. Mixed methods strengthen interpretation, but the quantitative component does not experimentally isolate infinite scroll. |
| 18 | Learning burnout and anxiety | Mao and Liao, 2025 | Chinese college students; **N = 523** valid questionnaires; convenience sampling; cross-sectional survey; regression mediation and PROCESS Model 5 | SVA was associated with learning burnout and anxiety; anxiety mediated the relationship, and negative coping moderated it. | Direct SFV | Component-consistent | Relevant to maintenance-stage impairment and affective burden. Cross-sectional moderated-mediation analysis cannot establish causal direction. |
| 19 | Attention control, learning burnout, and perceived writing competence | Chen et al., 2026 | Chinese university students; N = 559; cross-sectional survey; structural equation modeling | Short-video addiction was associated with decreased attention control and learning burnout, which were in turn associated with lower perceived English-writing competence. | Direct SFV | Component-consistent | Relevant to attentional control and functional academic outcomes. Cross-sectional self-report and mediation estimates do not establish causal ordering or measure recommendation exposure. |
| 20 | Usage intensity, perceived mood enhancement, and attention control | Ye et al., 2025 | Chinese adolescents; N = 1086; cross-sectional survey; structural equation modeling | Short-video usage intensity was positively associated with short-video addiction, perceived mood enhancement, and decreased attention control. | Direct SFV | Component-consistent | Relevant to use intensity, affective reinforcement, and attentional control. The cross-sectional design cannot establish temporal or causal direction, and the ‘TikTok brain’ construct remains preliminary. |
| 21 | Self-regulated learning and learning well-being | Ye et al., 2024 | Chinese undergraduate students; N = 706; cross-sectional online survey; structural equation modeling | Short-video addiction was negatively associated with preparatory, performance, and appraisal self-regulated learning strategies and indirectly associated with lower learning well-being. | Direct SFV | Component-consistent | Relevant to regulatory flexibility and functional outcomes. Cross-sectional mediation cannot establish that short-video addiction precedes changes in learning regulation or well-being. |
| 22 | Learning engagement and perceived learning ineffectiveness | Ye et al., 2023 | Chinese vocational students; N = 1,089; cross-sectional survey; structural equation modeling | Short-video problematic use was negatively associated with behavioral engagement; engagement dimensions were related to perceived learning ineffectiveness. | Direct SFV | Component-consistent | Relevant to academic functioning and sustained engagement. Self-report and cross-sectional modeling do not establish causal direction or isolate platform mechanisms. |
| 23 | Learning motivation and learning well-being | Ye et al., 2022 | Chinese vocational college students; N = 517; cross-sectional survey; structural equation modeling | Short-video flow was associated with addiction, while addiction was negatively associated with intrinsic and extrinsic learning motivation and indirectly associated with learning well-being. | Direct SFV | Component-consistent | Relevant to flow, motivation, and functional outcomes. Cross-sectional self-report does not establish the proposed temporal sequence or algorithmic contribution. |
| 24 | Engagement optimization and reflective welfare | Kleinberg et al., 2024 | Analytical/theoretical model of media consumption with inconsistent preferences and engagement optimization | Engagement-maximizing choices may diverge from reflective user welfare depending on movement through the content space. | Adjacent algorithmic | Boundary evidence | Provides a formal basis for distinguishing engagement from reflective welfare and for treating optimization effects as conditional rather than uniformly harmful. Not an SFV field study. |
| 25 | Algorithmic matching and endogenous interaction | López Vargas et al., 2022 | Large-scale field experiment in a digital social game; algorithmic assortative matching experimentally introduced | Algorithmic matching increased engagement and social interaction while producing greater segregation. | Adjacent algorithmic | Component-consistent | Demonstrates that algorithmic matching can causally alter interaction environments and behavior. The setting is a mobile social game rather than an SFV platform and does not test informational narrowing or PSVU directly. |
| 26 | Negative evaluation with continued use | Runge, 2025 | Large-scale observational mobile-gaming data combining behavioral clickstream measures with repeated reflective Net Promoter Score evaluations | Some users continued or increased consumption despite negative evaluations. | Adjacent behavioral | Component-consistent | Provides an operational example of behavior–evaluation discrepancy. Observational mobile-gaming evidence requires validation before transfer to SFV use. |
| 27 | Science-based player protection | Hayer and Kalke, 2022 | Systematic review; six electronic databases plus hand searching; **22 primary studies** evaluating **26 player-protection measures** | Personalized feedback, pre-commitment, and self-exclusion showed preventive potential, with modest effects and limited durability evidence. | Adjacent intervention | Mixed | Relevant to mechanism-matched digital safeguards and intervention design. Evidence derives from online gambling and cannot be directly generalized to SFV platforms. |

**Note.** This evidence map summarizes empirical and analytical studies relevant to individual components of the RNS framework. “Direct SFV” denotes studies conducted specifically in short-form video contexts, whereas “Adjacent” denotes evidence from related algorithmic, behavioral, or intervention domains. Most studies examine isolated associations or mechanisms rather than the complete recursive sequence. Cross-sectional mediation or structural-equation models should therefore not be interpreted as establishing causal ordering, and convergence across component-level studies should not be taken as validation of the integrated RNS framework.

**Supplementary Search Log**

The search strategy was designed to support an integrative conceptual review rather than an exhaustive systematic review. Records were assessed for their relevance to the proposed mechanisms, boundary conditions, measurement strategies, intervention targets, and countervailing or null evidence.

| **Database or tool** | **Core search blocks** | **Screening and use** |
| --- | --- | --- |
| PubMed | Short-form video or TikTok or Douyin or reels; problematic or addiction-like use; cognition, affect, automaticity, and intervention terms | Prioritized empirical and review articles directly addressing SFV use; retained adjacent-domain evidence only when it clarified a specified RNS mechanism or measure. |
| Crossref | Title and bibliographic searches combining platform terms with recommendation, personalization, diversity, narrowing, reinforcement, metacognition, data donation, and intervention terms | Used to identify and verify cross-disciplinary records and citation metadata; relevance was judged from title, abstract, and full text where available. |
| Google Scholar | Combinations of SFV terms with recommendation systems, serendipity, filter bubbles, processing fluency, cognitive load, automaticity, algorithmic literacy, digital intervention, and player protection | Used for multidisciplinary discovery and forward/backward citation chaining. Supportive, mixed, null, countervailing, and boundary-condition evidence was eligible; purely descriptive or mechanism-irrelevant records were excluded. |
